# Efficiency of diode laser in control of post-endodontic pain: a randomized controlled trial

**DOI:** 10.1007/s00784-023-04864-z

**Published:** 2023-01-20

**Authors:** Hend H. Ismail, Maram Obeid, Ehab Hassanien

**Affiliations:** Dept of Endodontics, Faculty of Dentistry, Ain Shams University, Cairo, Egypt

**Keywords:** Low-level laser therapy, Postoperative endodontic pain, Laser-activated irrigation, VAS

## Abstract

**Objectives:**

As post-endodontic pain control is one of the main targeted addressed in endodontics, our aim was delignated to compare two different methods for low-level laser application utilizing diode laser: low-level laser therapy (LLLT) and laser-activated irrigation (LAI) in the control of post-endodontic pain.

**Materials and subjects:**

A total of 180 patients received single-visit root canal treatment; they were randomly allocated into 3 equal groups. Group I received LAI, group II received LLLT, and group III served as control with normal root canal treatment and mock laser intervention (ML group). Postoperative pain was recorded using visual analogue scale (VAS) after 24, 48, and 72 h. Data were tabulated and statistically analyzed.

**Results:**

At 24 h, there was a statistically significant difference between median pain scores in the three groups (*P* value < 0.001) with ML group scored highest score followed by LAI and then LLLT group. At 48 h, there was a statistically significant difference between the three groups (*P* value < 0.001), with ML group scoring highest median pain scores while LLLT and LAI showed statistical insignificant scores. At 72 h, there was no statistically significant difference between the 3 groups (*P* value = 0.179).

**Conclusion:**

LLLT is superior to LAI and ML group in the control of immediate postoperative pain after 24 h while after 48 h both LAI and LLLT were equally effective, but they still showed significant differences when compared to ML group.

**Clinical relevance:**

Diode laser can be used by clinicians as it decreases the post-endodontic pain in patients with symptomatic apical periodontitis undergoing endodontic treatment.

## Introduction

The control of postoperative pain is a golden target with miscellaneous tactics to achieve as it is a frequent sequalae after endodontic treatment and is accredited to either mechanical, chemical, or microbial aspects [1]. The usage of analgesics namely nonsteroidal anti-inflammatory drugs (NSAIDs) is the most familiar way utilized to minimize this pain as they have a well-recognized pathway through the direct inhibition of cyclo-oxygenase enzymes 1 and 2 (COX) and inflammation [2]. Although their analgesic property is well understood, NSAIDs have been accompanied with numerous adverse effects, such as gastrointestinal bleeding [3].

Laser therapy has been employed in dentistry widely since its development by Maiman in 1960 [4]. Its usage had a beneficial effect on pain relief [5] and wound healing [6]. With the rapid improvements in its technology and increased knowledge about its interactions with tissues (bio-interaction), the clinical uses of laser in endodontics are broader nowadays [7–9]. The commonest form of that is the use of diode laser in a low-level laser therapy (LLLT) and to activate the root canal irrigants.

LLLT reacts with cells through light, a phenomenon called photo-biomodulation [8, 10]. It was reported that this has anti-inflammatory pain-relieving effect and considered as an aide to alleviate postoperative pains [5, 7, 11]. It was explained by the ability of low-level laser to modify pain threshold, lessen bradykinin, and histamine release, boost the formation of endogenous endorphins, and change prostaglandin production [12, 13].

Laser-activated irrigation (LAI) generate optical cavitation in the irrigant, causing the formation of vapor bubbles [14]. The laboratory studies of LAI have been revealed that it is very effective in debris removal from canal complexities [15] which occurs exceedingly fast, causing a very rapid turbulence in the liquid throughout the whole canal [16]. Moreover, LAI showed a reduced postoperative pain and discomfort following endodontic treatment in single visit [17, 18]. The latter was attributed to the irrigation activation’s low intra-canal pressure, which does not exceed the pressure in the apical tissues or the 5.88 mmHg central venous pressure [18].

As no comparison was found in literatures between these laser applications, this randomized controlled trial study aims to compare two different methods for low-level laser application: LLLT and LAI in terms of post-endodontic pain control in patients with symptomatic apical periodontitis undergoing primary root canal treatment. The null hypothesis assumes that there is no significant difference in postoperative pain after root canal treatment using LAI or LLLT.

## Materials and methods

### Study design and setting

**Fig. 1:**
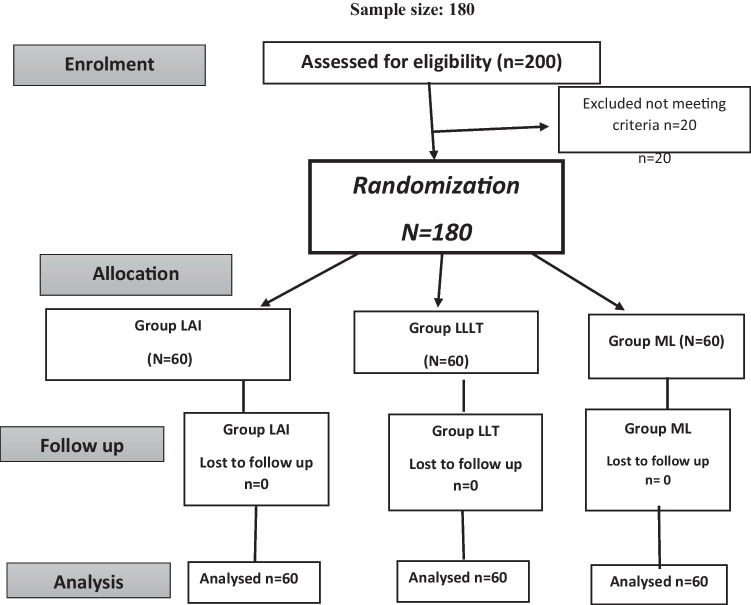
Consolidated standards of reporting trials flow diagram of the study

This study is a single blinded, three-arm, randomized, placebo-controlled, clinical trial that was designed and reported by adhering to the Consolidated Standards of Reporting Trials statement CONSORT [19]. A flow diagram representing the Consolidated Standards of Reporting Trials of the study is presented in Fig. 1. The study protocol was registered in the www.clinicaltrials.gov database with identifier number NCT04315259. It was assessed then approved by the Ethics committee, Faculty of Dentistry, Ain Shams University (Approval code: FDASURecID091703).

The study population was recruited from the outpatient Endodontic clinic at Faculty of Dentistry, Ain Shams University, between July 2017 and January 2020. All applicants signed a consent after a detailed description of the study’s aim, methods, advantages, and possible hazards.

### Sample size calculation

Sample size calculation was done using one-way analysis of variance power calculation for more than two groups in R statistical package, version 3.3.1, copyright (C) 2016, considering the pain described with the visual analogue scale (VAS) as the primary outcome. The expected effect size was calculated according to Yoo et al. [20]. We used a more conservative effect size of 0.3 instead of the estimated 0.59. We also added 25% compensation for potential non-responders. The results showed that, at a power of 90% and a two-sided significance level of 5%, a total sample size of 180 participants (equally allocated to three groups) will be adequate to detect an effect size of 0.3.

### Eligibility criteria

Medically free male patients aged between 18 and 50 years, suffering from acute or chronic infection with symptomatic apical periodontitis related to mandibular permanent molars with sensitivity to percussion and radiographic apical rarefaction, were enrolled in our study. Patients were rejected if they suffered from any medical problem or took any medication that could modify pain assessment (e.g., analgesics) within 12 h before the visit.

### Diagnosis and patient acceptance

An experienced endodontist (IH) (principal investigator) handles every aspect of dental treatment. After detailed medical and dental history, clinical and radiographic assessment was done to establish accurate diagnosis. Every participant was questioned to record the degree of preoperative pain by drawing a line on a VAS (0–10 cm), with 0 meaning “no pain” and 10 “intolerable pain.”

### Root canal treatment

Local anesthetic (1:80,000 Arcaine, Aarge Pvt Ltd., India) was administered, and endodontic access was achieved under rubber dam isolation. A periapical radiograph was used to confirm the working length that had been determined using an apex locator (Root ZX mini, J. Morita, Japan). Following the manufacturer’s specifications, canals were mechanically prepared using an engine-driven rotary nickel titanium Protaper Next system (Dentsply Maillefer, Ballaigues, Switzerland) with finishing files ranging from F1 to F5, depending on the canal’s original diameter. Using a torque-controlled endodontic motor (X-Smart; Dentsply Sirona) and the SX tool (ProTaper Universal; Dentsply Sirona, Ballaigues, Switzerland), the coronal section of the canals was enlarged. Each canal’s final apical preparation size was selected three sizes larger than the first binding file at the WL with a minimum apical diameter of size 25. Irrigation was accomplished using a 27-gauge needle attached to a 3-mL syringe (Kendall; Covidien, Mansfield, MA, USA) with 2 mL of 2.5% NaOCl in between files. The needle was advanced to a depth of 2–3 mm short of WL. The final irrigation was done using 2-mL EDTA 17% (Denteck, Zoetermeer, Netherlands) followed by 3 mL of normal saline.

### Randomization

After that, patients were randomly assigned to one of the three groups based on the mode of use of diode laser: group 1 (LLLT), group 2 (LAI), and group 3 (mock laser (ML)). The investigator created 18 envelopes containing concealed group codes to be assigned sequentially to the eligible patients. The operator opened the envelopes and recorded the codes with respective patient details on a computer to have adequate randomization using an equal proportion randomization allocation ratio.

### Intervention

- For patients in low-level laser therapy (LLLT) group, laser applicator was positioned 3 mm away from the oral mucosa with beam focused on apical location. Briefly, after the application of the final flush and with the aid of a dental applicator, a 200-mm fiber optic laser tip was placed perpendicular to soft tissue mucosa at the level of the apices of the targeted tooth based on the tooth length taken by the apex locator and confirmed by the working length periapical X-ray. Diode laser exposure (Lite Medics 1.00-Watt serial number 148 Ver. SwvM. 150VS108VT.100) was carried out using a continuous-mode with 10 Hz frequency, 980 nm wavelength, and max power 15 WCW. The tip was applied both at the buccal and the lingual side for 30 s each [21].
- For patient in laser-activated irrigation (LAI) group, at the step final step of irrigation, the irrigant was activated with diode laser (Master lase/expert lase, Kavo, Biberach/Riß) (980 nm wavelength and 2.5 W power, frequency of 100 Hz). The optical fiber was introduced 2–3 mm short of the working length and was withdrawn from apical to coronal direction [22]. Each canal was irradiated for 20 s, recurring three times with pauses of 10 s between each. The total exposure time was 60 s [23].
- For mock laser (ML) group: the diode laser tip was positioned similarly as in group 1 without activation (mock laser).

After root canal preparation, the root canals were dried with paper points and filled using matched single cones (Meta Biomed Korea) and AD seal sealer (Meta Biomed Korea) was used. Obturation was done using warm vertical compaction.

### Assessment of postoperative pain

All participants had a chart to record their pain after 24, 48, and 72 h of treatment. A visual analogue scale (VAS) (0–10 cm) was used to indicate the pain intensity where 0 = no pain and 10 = unbearable pain in Arabic. It was fully described to the participants, and they were instructed to return the reports at their next visit to the faculty. Additionally, participants were questioned orally about their use of antibiotics and analgesics over the previous 72 h when they turned in their VAS questionnaire and were excluded and replaced if they took medications. All data were collected and statistically analyzed. Recall rate of patients was 100% as any patient who failed to give feedback or took any analgesics was replaced by new cases.

### Statistical analysis

All data were gathered and organized in Excel sheets (Microsoft Corporation, Redmond, WA, USA). Demographic data in the three groups were compared utilizing one-way ANOVA test and Chi-square test. Various time interval within each group were analyzed using Friedman’s test and the comparison between groups was done utilizing Kruskal–Wallis test (significant at *P* ≤ 0.05).

## Results

There was no statistically significant difference between mean age values in the three groups presented in Table 1.

**Table 1:** Mean, standard deviation (SD), (*n*), and results of one-way ANOVA test and Chi-square test for comparisons of age in the three groups

	LAI group (*n* = 60)	LLLT group (*n* = 60)	ML group (*n* = 60)	*P* value
Age mean (SD)	36.3 (8)	38 (9.6)	37.6 (9.5)	0.566

^*^Significant at *P* ≤ 0.05

### Assessment of postoperative pain within each group

**Fig. 2:**
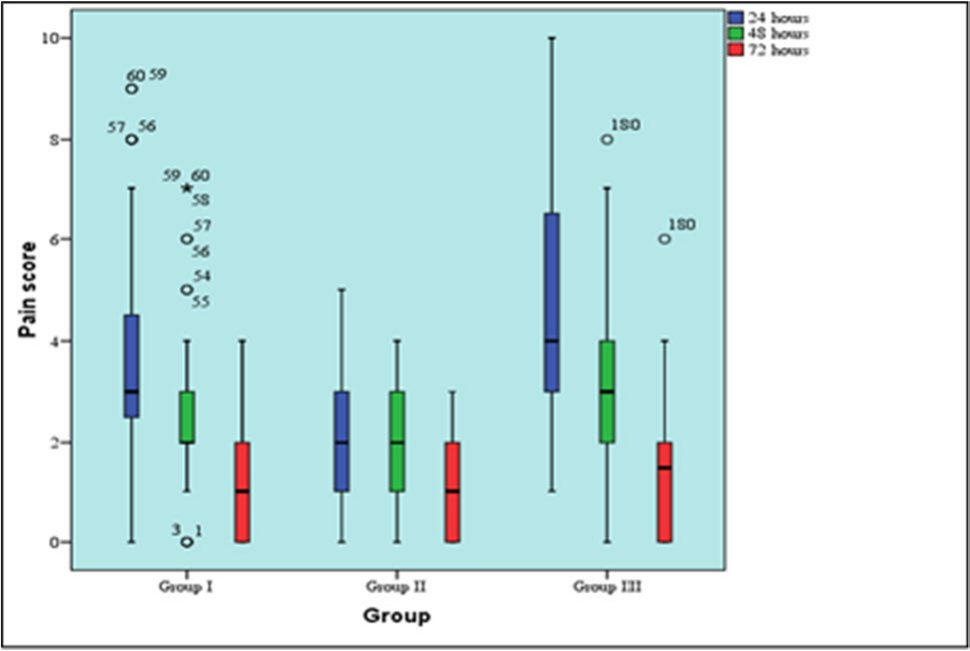
Box plot representing median and range values for pain scores in the three groups (stars and circles represent outliers)

All data are presented in Table 2 and Fig. 2. In LLLT group, there was a statistically significant change in pain scores by time (*P* value < 0.001, effect size = 0.725). In pairwise comparisons, statistically there was no significant change between median pain score after 24-to-48-h time intervals, but a statistically significant change recorded after 72 h. Results showed a decrease in median of pain score from 24 (median 2, range 0–5; *P* value < 0.001) to 48 h (median 2, range 0–4; *P* value < 0.001) and the least recorded was after 72 h (median 1, range 0–3; *P* value < 0.001).

**Table 2:** Median and mean (SD) of pain scores in the three groups at different time intervals

Time		LAI group	LLLT group	ML group	*P* value^1^	*Effect size (Eta squared)*^*1*^
24 h	Median (range)	3 (0–9) ^B a^	2 (0–5) ^C a^	4 (1–10) ^A a^	< 0.001*	0.332
	Mean (SD)	3.8 (2)	2 (1)	4.8 (2.3)		
48 h	Median (range)	2 (0–7) ^B b^	2 (0–4) ^B a^	3 (0–8) ^A b^	< 0.001*	0.098
	Mean (SD)	2.7 (1.7)	1.8 (1)	3.2 (1.8)		
72 h	Median (range)	1 (0–4) ^c^	1 (0–3) ^b^	1.5 (0–6) ^c^	0.179	0.008
	Mean (SD)	1.3 (1.1)	1.1 (1)	1.6 (1.3)		
	*P* value ^2^	< 0.001**	< 0.001**	< 0.001**		
	*Effect size (w)*^*2*^	0.959	0.725	0.988		

Different superscripts in the same row and column are statistically significantly different

^*^, **Significant at *P* ≤ 0.05

^*^Represents significance of *P* value of Kruskal–Wallis test between 3 groups

^**^Represents significance for *P* value of Friedman’s test within each group

1Results of Kruskal–Wallis test comparing the 3 groups together

2Results of Freidman’s test for changes by time within each group

In LAI group, there was a statistically significant change in pain scores by time (*P* value < 0.001, effect size = 0.959). In pairwise comparisons, statistically there was a significant change in pain scores from 24 to 48 h interval as well as from 48 to 72 h. Results showed a decrease in the median of pain scores from 24 (median 3, range 0–9, *P* value 0.001) to 48 h (median 2, range 0–7, *P* value < 0.001) and the least was recorded at 72 h (median 1, range 0–4, *P* value < 0.001).

In placebo group, there was a statistically significant change in pain scores by time (*P* value < 0.001, effect size = 0.988). In pairwise comparisons, statistically there was a significant change in pain scores recorded from 24 to 48 h’ time interval as well as from 48 to 72 h. Results showed a decrease in median pain scores from 24 (median 4, range (1–10), *P* value < 0.001) to 48 h (median 3, range (0–8), *P* value < 0.001)) and the least recorded was after 72 h (median 1.5, range (0–6), *P* value < 0.001).

### Assessment of postoperative pain between the three groups

After 24 h, there was a statistically significant difference between median pain scores in the three groups (*P* value < 0.001, effect size = 0.332). Pairwise comparisons between the groups revealed that ML group showed the statistically significantly highest median pain score (median = 4, range (1–10), *P* value < 0.001) followed by LAI group (median 3, range (0–9), *P* value < 0.001), and the lowest pain score was in LLLT group (median 2, range (0–5), *P* value < 0.001).

After 48 h, there was a statistically significant difference between median pain scores in the three groups (*P* value < 0.001, effect size = 0.098). Pairwise comparisons between the groups revealed that ML group has statistically significantly highest median pain score (median 3, range 0–8, *P* value < 0.001). There was no statistically significant difference between LAI group and LLLT group; both showed the statistically significant lower median pain scores.

After 72 h, there was no statistically significant difference between median pain scores in the three groups (*P* value = 0.179, effect size = 0.008).

## Discussion

Varying levels of pain and discomfort may persist even after removal of the inflammation source by cleaning and shaping procedures [1] and since analgesics causes a lot of undesirable side effects [2, 3], it was necessary to investigate other methods to relief pain.

The use of laser in low level had a notable effect on the outcomes of endodontic treatment; our point of search focused to investigate the optimum low-level laser intervention methodology that may affect the postoperative pain level. Thus, our aim was to compare two different methods for low-level laser applications: LLLT and LAI in terms of post-endodontic pain control.

Our study is a single blinded, three-arm, randomized, placebo-controlled, clinical trial with patient-reported outcome procedures for the greatest possible chance to minimize bias and in which the patient, who is the outcome reporter, was blinded to the interventions done, consequently decreasing act and find bias [19].

Only persons who did not take any pain modulating medications 12 h before the visit were allowed to participate in the study to abolish the influence of pre-treatment analgesics on pain analysis. The impact of gender on postoperative pain has been conflicting [24]; thus, our study targeted male patients only for standardization. It is noted that harsh flare-ups and severe pain have been correlated with symptomatic apical periodontitis and that the presence of preoperative periapical radiolucency is associated with pain lasting more than 2 days [25]. And as molars usually came up with the greatest frequency of postoperative pain [26], consequently, symptomatic apical periodontitis with radiographic apical rarefaction related to mandibular permanent molars were addressed to mimic the worst situation imaginable.

A single-visit root canal treatment was selected in this study to remove any positive or negative influence of using intracanal medication on our outcome [27]. To avoid debris or irrigant extrusion beyond the apical foramen, the needle penetration depth was maintained 3 mm shorter than the WL [28]. A rotary system followed the manufacturer’s recommendations and was used similarly with all participants. Mock laser was used to function as a placebo that is widely utilized in the medical field for clinical studies as it enables blinding [29].

As studies reported elevated levels of post-endodontic pain after 48–72 h [30, 31], our time window frame was 24, 48, and 72 h. We did not record pain level scores before 24 h to make sure that the anesthetic effect does not interfere with the patient’s actual sensation of pain as suggested by Flath et al. [32].

Regarding the choice of the wavelength and the power of the diode laser in our study, we used wavelength of 980 because as it showed more bactericidal with signs of changes in the bacterial surface [22]. The used power was low to have a beneficial therapeutic effect rather than harmful effect accompanied high-intensity lasers in the form of thermal coagulation of tissue [33].

Proper positioning of the fiber optic probe in LAI was mastered to be 2–3 mm short of the WL. This was to ensure adequate irrigant flow through the root canal [17] and to aid in avoiding the extrusion of irrigant and debris. This fiber was moved slowly up to maximize its effect [22].

In LLLT group, the laser beam was targeted directly over the alveolar mucosa at a location corresponding to the apices of each tooth, although the beam was targeted in other studies [34] 10 mm away from the tissues, and as we are using a laser beam of lower energy (1 W), we decided to place it in contact with the alveolar mucosa.

Our results showed that there was statistically significant difference in pain score after 24 h where lowest pain score was related to LLLT group. After 48 h, there was no statistically significant difference between LAI and LLLT groups, but both differ statistically significantly lower than ML group.

The results of our study agree with the findings of Arsalan A et al. [21] and Çakici E [35] where both concluded that low-level laser therapy reduced postoperative pain after single visit. LLLT is deemed as non-invasive therapies by light [7] embody the principle of phototherapy and contribute to the same concept of photobiomodulation [8]. LLLT impacts both local microcirculation and cellular metabolism [36]. It boosts the production of prostaglandins (PGI2) with its anti-inflammatory effects [37]. Moreover, LLLT prevents the production of pro-inflammatory factors and pain-related neurotransmitters [38] and encourages the elimination of pain-inducing substances (histamine, substance P, and dopamine) [39]. All these biological activities could make sense of the positive outcomes introduced in this study.

LAI showed favorable responses in pain control in several studies [18, 40–42]. We proofed that the strong irrigant dynamics triggered by LAI did not result in increasing the postoperative pain. This was in line with the RCT by Dagher et al. [41], who also did not find any significant difference in pain after endodontic treatment whether syringe irrigation or LAI used and coincide with the results of ex vivo researched proving that the extrusion of irrigant with LAI is equivalent to other irrigation methods [43].

Considering that both LAI and LLLT are clinically viable options, using LAI would be the more clinically useful and effective option given the similar results. This is because LAI has been shown to have antibacterial effects, is reasonably priced, and is increasingly used in general practice.

A limitation of this research may be the unenrolment of female patients; thus, wider range of patients may enhance the generalizability of the clinical trial outcomes. Upcoming studies are advised to assess the effects of other lasers on the short- and long-term results.

## Conclusion

LLLT is superior to LAI and ML group in the control of immediate postoperative pain after 24 h while after 48 h both LAI and LLLT were equally effective, but they still showed significant difference when compared to ML group. After 72 h LAI, LLLT and conventional root canal treatment had similar results in the control of post-endodontic pain.

## Data Availability

Both are available upon request.
